# Structure of the afferent terminals in the terminal ganglion of a cricket and persistent homology

**DOI:** 10.1186/1471-2202-13-S1-P134

**Published:** 2012-07-16

**Authors:** Jacob Brown, Tomas Gedeon

**Affiliations:** 1 Department of Mathematical Sciences, Montana State University, Bozeman, MT 59717, USA; 2 Center for Computational Biology, Montana State University, Bozeman, MT 59717, USA

## 

In many areas of biology there is an abundance of noisy and imprecise data, but often a lack of high precision quantitative data. It is therefore important to design, and use, data analysis methods that are insensitive to small errors in measurements. We present such a robust data analysis as we investigate the three dimensional spatial structure of the locus of afferent neuron terminals in crickets Acheta domesticus. Each afferent neuron innervates a filiform hair positioned on a cercus: a protruding appendage at the rear of the animal. The hairs transduce air motion to the neuron signal that is used by a cricket to respond to the environment. We stratify the hairs (and the corresponding afferent terminals) into four classes, which depend on hair length as well as the position of each hair. Our analysis uncovers significant structure in the relative position of these terminal classes and suggests the functional relevance of this structure. Our method is very robust to the presence of significant experimental and developmental noise. It uses ideas from persistent homology [1-3]. This method is applicable to any point cloud data set that exhibits an underlying topological structure corrupted by noise.

We show that each individual terminal class has similarly positioned significant tunnels and/or voids. However, while each class' point cloud is of similar shape and structure, the space that each class occupies is slightly offset, resulting in no significant tunnels and/or voids in the set of terminals of all hairs. This result is not surprising from an efficiency standpoint: the space in the terminal ganglion is limited and the terminals of afferent neurons are filling the entire available space. While, we emphasize that there are still visible tunnels and/or voids in the entire point cloud, our analysis provides us with the conclusion that these remaining tunnels are not significant, and very likely contain the dendrites of the interneurons in the terminal ganglion. These are downstream from the afferents, and make synaptic contact with the afferent terminal cloud.
